# Low-Level Vagus Nerve Stimulation Reverses Obstructive Sleep Apnea-Related Atrial Fibrillation by Ameliorating Sympathetic Hyperactivity and Atrial Myocyte Injury

**DOI:** 10.3389/fphys.2020.620655

**Published:** 2021-01-26

**Authors:** Yankai Guo, Jiasuoer Xiaokereti, Qingjun Meng, Guiqiu Cao, Huaxin Sun, Xianhui Zhou, Ling Zhang, Baopeng Tang

**Affiliations:** ^1^Department of Pacing and Electrophysiology, The First Affiliated Hospital of Xinjiang Medical University, Xinjiang, China; ^2^Xinjiang Key Laboratory of Cardiac Electrophysiology and Cardiac Remodeling, The First Affiliated Hospital of Xinjiang Medical University, Xinjiang, China; ^3^Department of Cardiology, The Fifth Affiliated Hospital of Xinjiang Medical University, Xinjiang, China

**Keywords:** obstructive sleep apnea, atrial fibrillation, low-level vagus nerve stimulation, sympathetic hyperactivity, atrial myocyte injury

## Abstract

**Background**: Previous studies have proved that low-level vagus nerve stimulation (LLVS) could suppress acute obstructive sleep apnea (OSA), which is associated with atrial fibrillation (AF).

**Objective**: This study investigates the underlying electrophysiological, neural, and cardiomyocyte injury mechanisms on acute OSA-induced AF, examining whether LLVS can attenuate or reverse this remodeling.

**Methods and Results**: Eighteen mongrel dogs received endotracheal intubation under general anesthesia and were randomly divided into three groups: the OSA group (simulated OSA with clamping of the trachea cannula at the end of expiration for 2min followed ventilation 8min, lasting 6h, *n*=6), the OSA+LLVS group (simulated OSA plus LLVS, *n*=6), and a control group (sham clamping the trachea cannula without stimulation, *n*=6). In the OSA+LLVS group, the atrial effective refractory period was significantly lengthened while the sinus node recovery time and AF duration decreased after the 4th hour, and the expression level of Cx40 and Cx43 was significantly increased compared to the OSA group. Norepinephrine, TH, and ChAT were significantly decreased in the OSA+LLVS group compared with the OSA group. Mitochondrial swelling, cardiomyocyte apoptosis, and glycogen deposition, along with a higher concentration of TNF-α, IL-6 were observed in the OSA group, and the LLVS inhibited the structural remodeling and expression of inflammatory cytokines.

**Conclusion**: LLVS decreased the inducibility of AF partly by ameliorating sympathetic hyperactivity and atrial myocyte injury after acute OSA-induced AF.

## Introduction

Obstructive sleep apnea (OSA), the most common and severe form of sleep disordered breathing, is an important potential risk factor for the initiation and maintenance of atrial fibrillation (AF; Gami et al., 2004; Szymański et al., 2014). Previous studies have shown that the prevalence of AF in OSA patients ranges from 32 to 49% (Todd et al., 2010), and the incidence of OSA is also higher in the AF population (Szymański et al., 2014). To date, several pathophysiological mechanisms have been shown to contribute to AF in chronic OSA, including hypoxia, intrathoracic pressure shifts, sympathovagal imbalance, neurohumoral activation, atrial remodeling, oxidative stress, inflammation, and among others (Zhang et al., 2015a).

Recently, increasing evidence shows that sympathovagal imbalance plays a crucial role in the maintenance of AF, and neuromodulation through rebalancing sympathovagal activity has become a research hotspot. Previous studies have shown that low-level vago-sympathetic trunk stimulation (LLVS) at voltages that do not slow the sinus rate or AV conduction, could decrease AF inducibility and AF duration, and prolonged ERP induced by acute OSA on a rabbit model (Gao et al., 2015), but no changes have yet been manifested in the structural remodeling of the left atrium (LA). Yu et al. (2017) demonstrated that noninvasive low-level transcutaneous electrical stimulation could prevent the incidence of AF in a 1-h OSA dog model. They also recorded neural activity from the superior left ganglionated plexus (SLGP), the left stellate ganglion (LSG), and the left renal sympathetic nerve (RSN), as well as changes in fast neuron markers in SLGP and LSG, but the nerve distribution in LA has not yet been investigated. In another study, Linz et al. (2012, 2013) showed reduced AF-inducibility and AERP shortening through renal sympathetic denervation and low-level baroreceptor stimulation in an acute OSA-induced AF pig model. The role of these treatments could inhibit AF by intervening in the activity of the autonomic nervous system (ANS). However, the relationship between neural activity and atrial myocyte injury has not been elucidated.

This study established a 6-h OSA model in dogs to examine changes in electrophysiological parameters, autonomic nervous activities, systemic inflammation, and myocardial damage and explore the possible mechanisms. In doing so, we aimed to provide underlying theoretical support for LLVS as a treatment for acute OSA-induced AF.

## Materials and Methods

All experiments were reviewed and approved by the Animal Use and Management Ethics Committee of the First Affiliated Hospital of Xinjiang Medical University (IACUC-20170706-09) and were confirmed to adhere to Guide for the Care and Use of Laboratory Animals published by the US National Institutes of Health (NIH Publication No. 85-23, revised 1996).

### Animal Preparation

A total of 18 healthy male mongrel dogs (weight, 18±4kg) were used in this investigation. Each animal was anesthetized *via* intramuscular injection with a mixture of Zoletil (0.1mg/kg; Virbac S.A. France) and xylazine (5mg/kg; Huamu Animal Health Care Products Co., Ltd., China), followed by sodium pentobarbital (50–80mg/kg) as needed every 2h to maintain anesthesia. We assessed the effect of anesthesia by observing the disappearance of the eyelash reflex and tongue drag resistance. All dogs were safe and stable throughout the experiments. Standard surface electrocardiography and arterial blood pressure were continuously monitored using a Lead-7,000 (Sichuan Jinjiang Electronic Technology Co., Ltd. China). At the end of the study, animals were euthanized and heart tissues were harvested for further examination.

### Experimental Protocol

**Figure 1:**
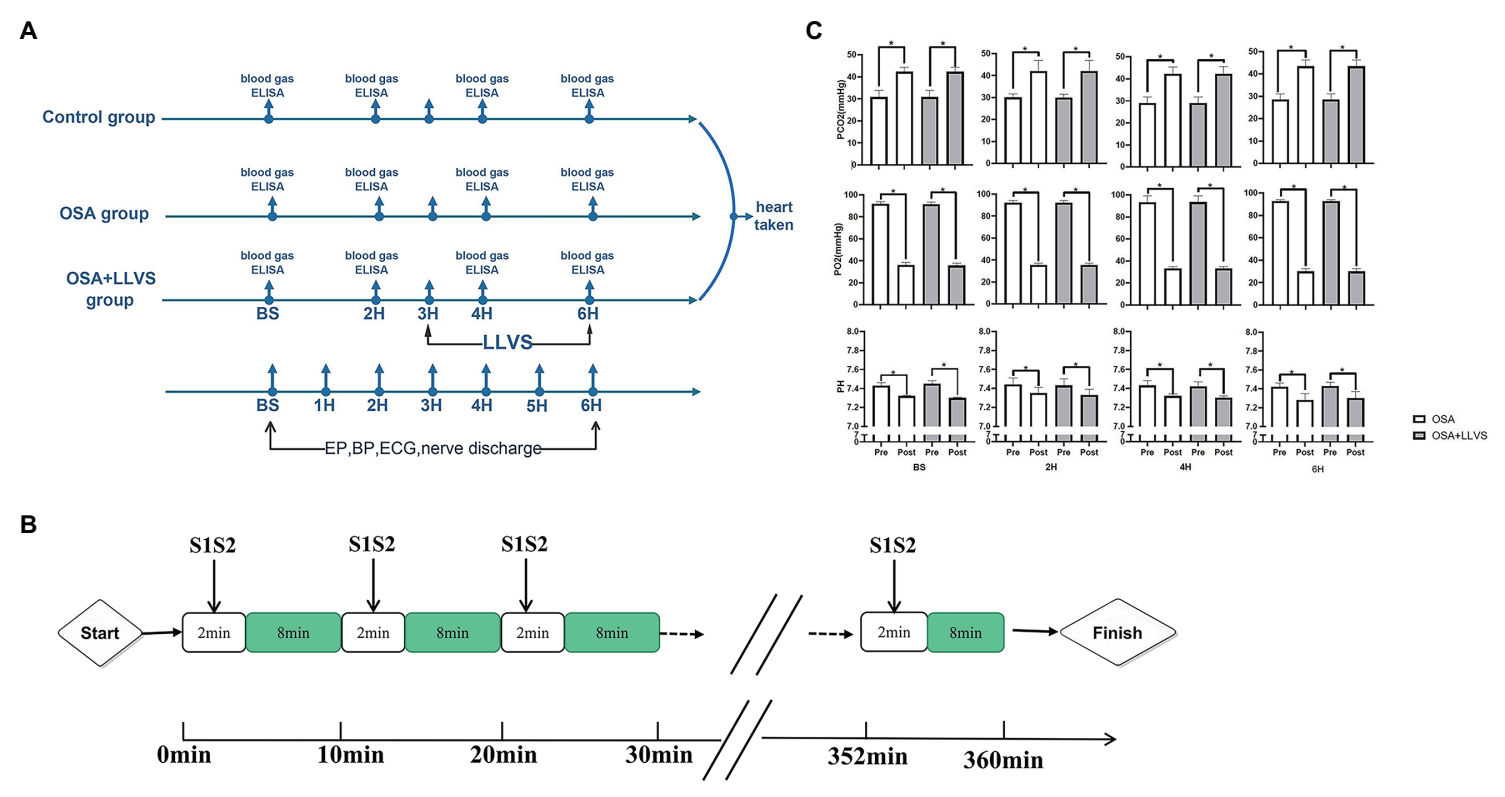
Flow chart of the study protocol. **(A)**. Acute OSA protocol. **(B)**. Changes in blood gases pre- and post-apnea between the OSA and OSA+LLVS groups. **(C)**. ^*^p<0.05 vs. the pre-apnea; OSA, obstructive sleep apnea; LLVS, low-level vago-sympathetic trunk stimulation; EP, electrophysiology; BP, blood pressure; ECG, electrocardiography; PaO_2_, partial pressures of oxygen; PaCO_2_, partial carbon dioxide tension; ELISA, enzyme-linked immunosorbent assay; H, hours; min, minute.

All animals were randomly divided into three groups: the OSA group (OSA for 6h, *n*=6), the OSA+LLVS group (OSA for 6h, of which the 4th to the 6th hour were accompanied by LLVS, at the voltage of 50% below of the slowing the sinus rate, *n*=6), and the control group (only intubated without clamping the tracheal cannula, *n*=6). Figure 1A illustrates the acute OSA protocol.

### Establishment of the Acute OSA Model

All animals under general anesthesia were intubated. The obstructive apnea model was established by clamping the tracheal cannula at the end of expiration for 2min, followed by 8min of ventilation (Ghias et al., 2009; Gao et al., 2015). This protocol for OSA was repeated every 10min for 6h (Figure 1B).

### Electrophysiological Study

A 7F catheter was inserted *via* the left femoral vein and the right jugular vein and inserted into an HRA electrode and a His electrode, respectively. The atrial effective refractory period (AERP), AF duration time, sinus node recovery time (SNRT), atrial-His interval (AH interval), and His-ventricle interval (HV interval) were measured at baseline and during the OSA process. AERP was measured by applying 8 consecutive S1 stimuli (S1-S1-330ms) followed by premature S2 stimuli with 10 times diastolic threshold, which was gradually decreased until capture no longer occurred. The SNRT, which indicated the function of the sinus node, was recorded between the last paced beat and the first spontaneous atrial depolarization (Zipes, 1992). The AF duration, AH interval, and HV interval were also determined (Figure 1A).

### LLVS Stimulation

The left vagosympathetic trunk was exposed by dissection and received continuous high-frequency electrical stimulation (HFS: 20Hz, 0.1ms duration, square waves) by a Grass stimulator (S88X, Astro-Med Inc., Warwick, RI). The threshold voltage was set to a voltage that did not slow the sinus heart rate or AV conduction (measured by the A-H interval; Yu et al., 2011). In this experiment, a 50% threshold voltage was administered for LLVS.

### Blood Gas Analysis

Arterial blood for blood gas analysis was drawn from the femoral artery *via* an anaerobic heparinized syringe at baseline, in the 2nd hour, the 4th hour, and the 6th hour (at the pre- and post-apnea event). The pH value, partial pressure of oxygen (PaO_2_), and partial carbon dioxide tension (PaCO_2_) were calculated using an i-STAT300 Analyzer (Abbott Laboratories, United States). All the arterial blood samples were analyzed within 10min of being collected.

### Blood Pressure Analysis

A blood pressure monitoring device was inserted into the left femoral artery. Blood pressure changes were recorded throughout the experiments. The systolic pressure of the arteries was analyzed. The blood pressure monitoring device was one part Lead-7000 (Sichuan Jinjiang Electronic Technology Co., Ltd. China).

### Neural Activity Recording

During the 6th hour of OSA, the left vagal nerve and LSG activity were recorded during the 2min of apnea and followed 40s of re-ventilation with PowerLab (Bio Amp; ADInstruments). The Analysis Module of Lab Chart 8.0/proV7 software (Bio Amp; ADInstruments) was used to analyze the signals. More detailed outlines of these processes are reported in our previous studies (Zhou et al., 2016; Zhang et al., 2018).

### Echocardiography

Transthoracic echocardiography was administered at baseline, the 3rd hour, and the 6th hour with a phased-array system (Sonos5500, Philips Ultrasound, United States). The following parameters were recorded: left atrial end-diastolic diameter (LA), right atrial end-diastolic diameter (RA), and left ventricular fractional shortening (FS). Each measurement used an average of three consecutive cardiac cycles.

### Enzyme-Linked Immunosorbent Assay

Venous blood was drawn at baseline in the 3rd hour and the 6th hour to measure the concentration of norepinephrine (NE; KA1877, Novus, CO, United States), TNF-α (HSTA00E, Novus, CO, United States), and IL-6 (D6050, Novus, CO, United States) using ELISA kits, respectively. The detection procedure was performed according to the manufacturer’s protocols.

### Tissue Staining

At the end of the study, left atrial (LA) tissues were harvested and fixed in 10% neutral buffered formalin for 24h at 4°C. They were then dehydrated, embedded in paraffin and consecutively cut at 5-μm thicknesses, and mounted on glass. These sections were used for staining with HE, PAS, TUNEL, and silver following the manufacturer’s protocols (Western Biomedical Technology, Hubei, China). A microscope (Zeiss, Germany) was used to study histological changes. The area of the interstitial fiber was calculated using image analysis software (Image-Pro Plus: IPP 7.0, Meida Cybernetics LP). The apoptotic rate of cardiomyocytes was calculated by the number of apoptotic cells/total cell number ×100%. The nerve density was calculated as positive nerve area/total area ×100%.

### Transmission Electron Microscopy

To detect ultrastructural changes in LA and LSG, transmission electron microscopy (JEM-1220, JEOL Ltd., Tokyo, Japan) was constructed. Briefly, tissues were processed by being fixed (2% glutaraldehyde and 2% paraformaldehyde), stained (1% uranyl acetate), dehydrated (ethanol), and embedded (epoxy resin).

### Immunohistochemistry

Immunohistochemistry staining was performed to evaluate the nerve fiber density of the LA tissues. Paraffin-embedded tissue sections were incubated with anti-TH (tyrosine hydroxylase, LS-C354110, 1:100, LifeSpan BioSciences, Seattle, WA, United States), anti-ChAT (choline acetyltransferase, LS-C79271, 1:100, LifeSpan BioSciences, Seattle, WA, United States), and anti-PGP9.5 (1,50, LS-B6518, LifeSpan BioSciences, Seattle, WA, United States) antibodies overnight at 4°C. After being washed with PBS, the sections were incubated with peroxidase-conjugated rat anti-rabbit IgG (LS-C60921, LifeSpan BioSciences, Seattle, WA, United States) for 20min at room temperature, and they were finally evaluated with a microscope at ×200 magnification (Leica, Wetzlar, Germany). Image Pro Plus 6.0 software (Media Cybernetics, United States) was used to analyze the image. Nerve fiber density was calculated as the ratio of positive nerve area to the total area (μm^2^/mm^2^). Connexin-40 (Cx40, LS-B959, 1:100, LifeSpan BioSciences, Seattle, WA, United States), and connexin-43 (Cx43, LS-B9771, 1:100, LifeSpan BioSciences, Seattle, WA, United States) were also detected, with quantitative analysis of integrated optical density (IOD) also conducted.

### Quantitative Real-Time Polymerase Chain Reaction (qRT-PCR)

At the end of the experiment, all dogs were euthanized, and the left atrial tissues were obtained. The mRNA expression levels of ion channels were measured by qRT-PCR. Total RNA was extracted using TRIzol reagent (Invitrogen, WA, United States) and then reverse-transcribed to cDNA using a PCR Kit (Invitrogen, WA, United States) according to the manufacturer’s instructions. An RT-PCR amplification reaction was performed as follows: 55 amplification cycles of 10s at 95°C, 20s at 58°C, and 20s at 72°C. The specificity of amplification was confirmed by melting curve analysis. A housekeeping gene (β-actin) was used to correct the expression level of the target gene, and the calculation was performed by a comparative method (2^−*Δ*ΔCt^). The primers used for RT-PCR are presented in Table 1.

**Table 1:** Primers used in RT-PCR.

Primer	Forward	Reverse
Nav1.5	GCATGGCTAACTTCGCTTATG	AGGATGGGGCTGAGGAGG
Cav1.2	AACTTTGACAATTTTGCCTTCG	AACTCATAGCCCATAGCGTCC
Kir2.1	TGGGAACGGGAAGAGTAAGG	ACGAACGCCAGGCAGAAG
Kir3.1	CTGGTGGACCTCAAGTGGC	GGGCTTTGTTCAGGTCGC
Kir3.4	ATCTGGTGGCTGATTGCTTACA	ATGATGCCCTCTGGACACTTC
HCN4	GAACAACTCCTGGGGAAAGC	CCGATGAACATGGCGTAGC

### Statistical Analysis

All continuous data were presented as the mean±standard deviation (SD) and analyzed using SPSS 19.0 software. Two-way repeated-measures ANOVA with *post hoc* Tukey test for three group variances. A paired *t* test was used to compare blood gas values under the baseline condition and after apnea. Statistical significance was defined at *p*<0.05.

## Results

### Arterial Blood Gas Analysis

To validate the OSA model, we analyzed changes in blood gas levels pre- and post-apnea for 2min at baseline, in the 2nd hour, the 4th hour, and the 6th hour. There were no significant differences in characteristics of pre-apnea between the OSA and OSA+LLVS group (*p*>0.05). A marked reduction in PaO_2_, with obvious increases in PaCO_2_ and pH, were observed post-apnea (*p*<0.05). The alterations were not affected by LLVS intervention. The above experimental data indicated that the OSA model was successfully established (Gao et al., 2015; Figure 1C).

### Effect of LLVS on ANS Activity in Acute OSA

**Figure 2:**
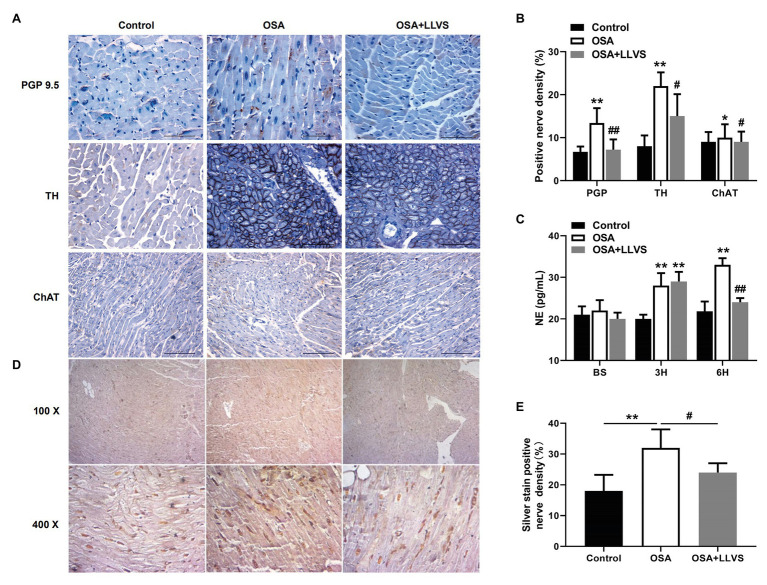
**(A)** Representative immunohistochemical staining of PGP 9.5, TH, and ChAT on the LA among the three groups. **(B)** Quantitative analysis of PGP 9.5, TH, and ChAT-positive nerve density in the LA of each group. **(C)** The concentration of NE among the three groups at baseline, the 3rd hour, and the 6th hour. **(D)** Representative silver staining in the LA among the three groups. **(E)** Quantitative analysis of silver staining-positive nerve density in the LA of each group. ^*^p<0.05, ^**^p<0.01 vs. the control group; ^#^*p*<0.05, ^##^*p*<0.01 vs. the OSA+LLVS group; PGP 9.5, protein gene product; TH, tyrosine hydroxylase; ChAT, choline acetyltransferase; NE, norepinephrine; H, hours.

Changes in autonomic nerve activity were analyzed to detect the expression levels of PGP 9.5, TH, and ChAT in LA tissues and the concentration of NE in serum. Higher expression levels of PGP 9.5, TH, and ChAT-positive nerve densities and a higher concentration of NE were observed in the OSA group compared with the control group, which were reversed by LLVS treatment during the final 3h (Figures 2A–C). Moreover, the hyperinnervation of nerve fibers in LA was evaluated through silver staining. Compared with that of the control group, the distribution of nerve fibers in LA was significantly increased, which was attenuated by LLVS treatment (Figures 2D,E). Additionally, changes in stellate ganglion were detected by transmission electron microscopy, part of the myelinated nerve fibers in the left stellate ganglion in the OSA group was swollen and degenerated, and the number of myelin sheaths in the OSA+LLVS group was reduced (Figure 6F). The above results indicated that LLVS could modulate the autonomic nerve imbalance in acute OSA-induced AF.

### Changes in Sympathovagal Activity, BP, and HR During a Cycle of Apnea and Re-Ventilation

During a cycle of apnea and re-ventilation, changes in sympathovagal activity were recorded at the apnea of 40, 80, 120s, and re-ventilation of 40s. At the first 40s of apnea, the activity of the sympathetic was enhanced, and the activity of the parasympathetic changed a little (Figure 4Aa). At the second 40s of apnea, the activity of the sympathetic was increasingly enhanced, and the activity of the parasympathetic became overdriving (Figure 4Ab). At the third 40s of apnea, the amplitude of sympathetic nerve firing increased significantly, while the frequency decreased, and the vagus nerve firing increased significantly, but the amplitude was significantly lower than that of the sympathetic nerve, the lower BP, slower HR was observed at the same time (Figures 4Ac,C). At the first 40s of re-ventilation, the frequency of sympathetic nerve firing increased and the amplitude decreased, while the vagus nerve continued to be in a state of high firing, manifesting higher BP and faster HR (Figures 4Ad,C).

**Figure 3:**
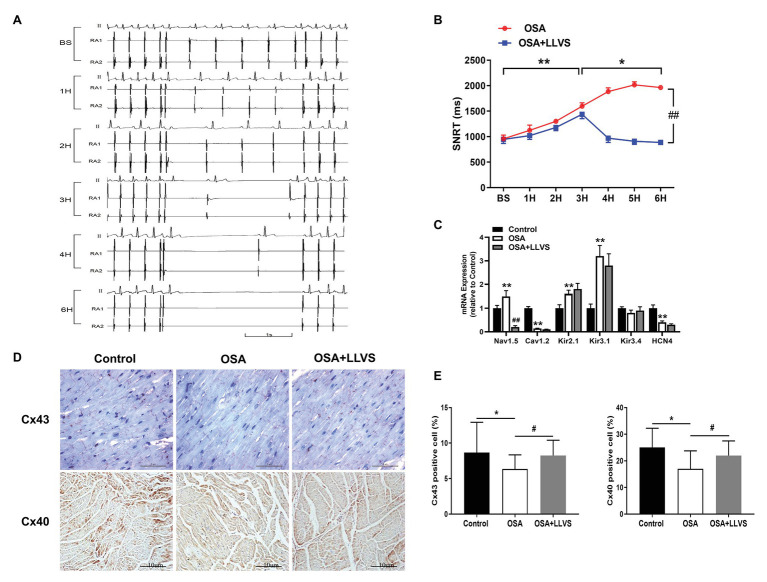
**(A)** Representative examples of SNRT in the OSA group at baseline, the 1st hour, the 2nd hour, the 3rd hour, the 4th hour, and the 6th hour. **(B)** Changes in SNRT between the OSA and OSA+LLVS groups at each hour. **(C)** Changes in ion channels *via* gene expression level among the three groups. **(D)** Representative immunohistochemical staining of Cx 40 and Cx 43 on the LA among the three groups. **(E)** Quantitative analysis of Cx 43 and Cx 40-positive cells in the LA of each group. ^*^p<0.05, ^**^p<0.01 vs. the control group; ^#^*p*<0.05, ^##^*p*<0.01 vs. the OSA+LLVS group; SNRT, sinoatrial node recovery time; Cx 43, connexin 43; Cx40, connexin 40; H, hours.

**Figure 4:**
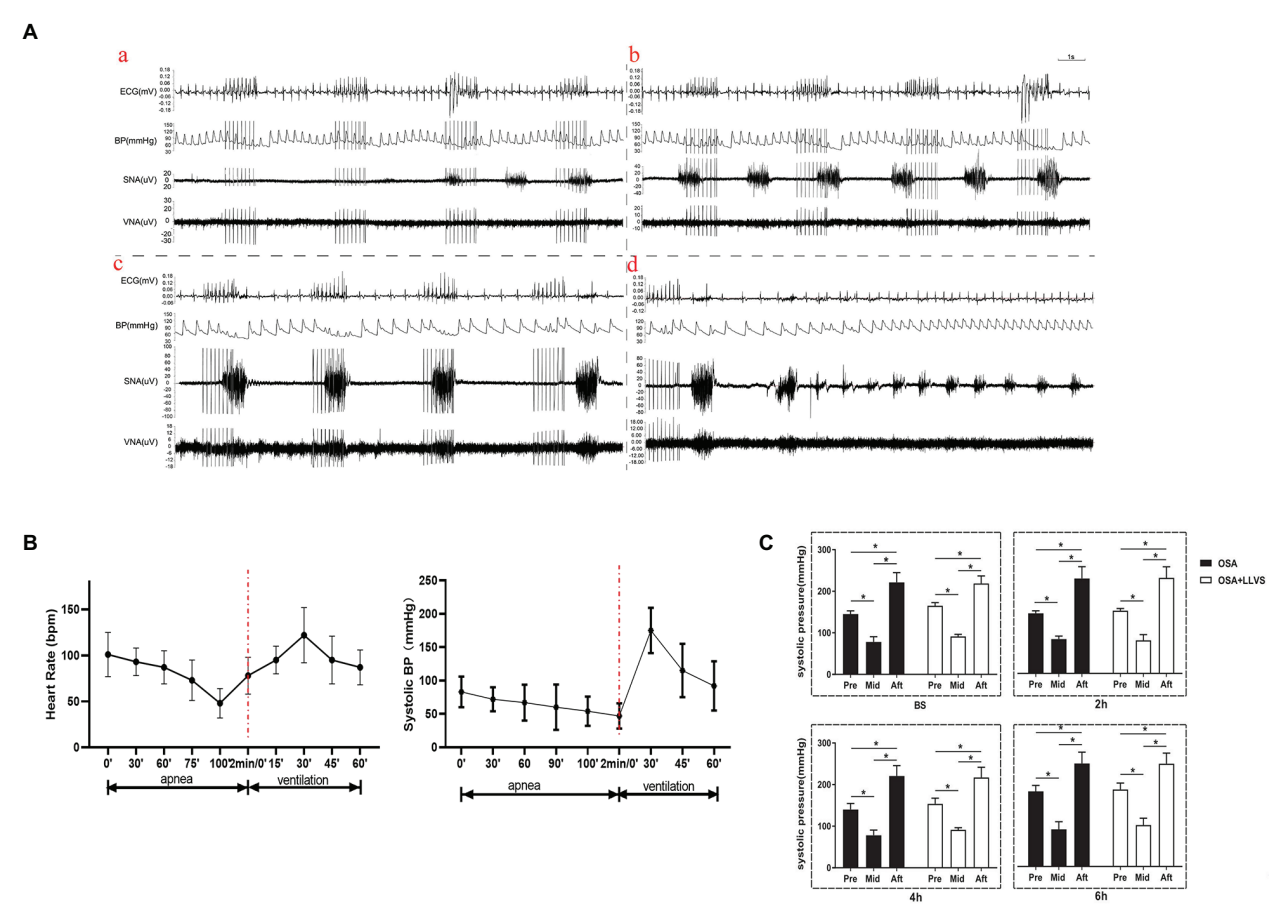
**(A)** Neural activity recording during the process of apnea and re-ventilation, **(a)** representative examples of the neural recording at the first 40s of apnea, (b) representative examples of the neural recording at the second 40s of apnea, (c) representative examples of the neural recording at the third 40s of apnea, (d) representative examples of the neural recording at the first 40s of re-ventilation; **(B)** The dynamic changes in HR and BP before and after apnea in OSA; **(C)** Changes in BP pre and after apnea between the OSA and OSA+LLVS groups at baseline, the 2nd hour, the 4th hour, and the 6th hour. ^*^p<0.05. BS, baseline; H, hours. HR, heart rate; BP, blood pressure.

Within 2min of apnea, both BP and HR also gradually decreased. Once re-ventilation was initiated, BP and HR gradually increased from 0 to 30s, after which the changes gradually decreased and approached baseline levels during the 30–60s (Figure 4B).

These data indicate that both the parasympathetic and sympathetic activity were overdriving in the acute apnea, revealing that the homeostasis of the sympathetic nerve and vagus nerve underwent dramatic changes once the apnea interval switched to ventilation, which subsequently promoted the incidence of AF.

### Effect of LLVS on Electrical Remodeling in Acute OSA

**Figure 5:**
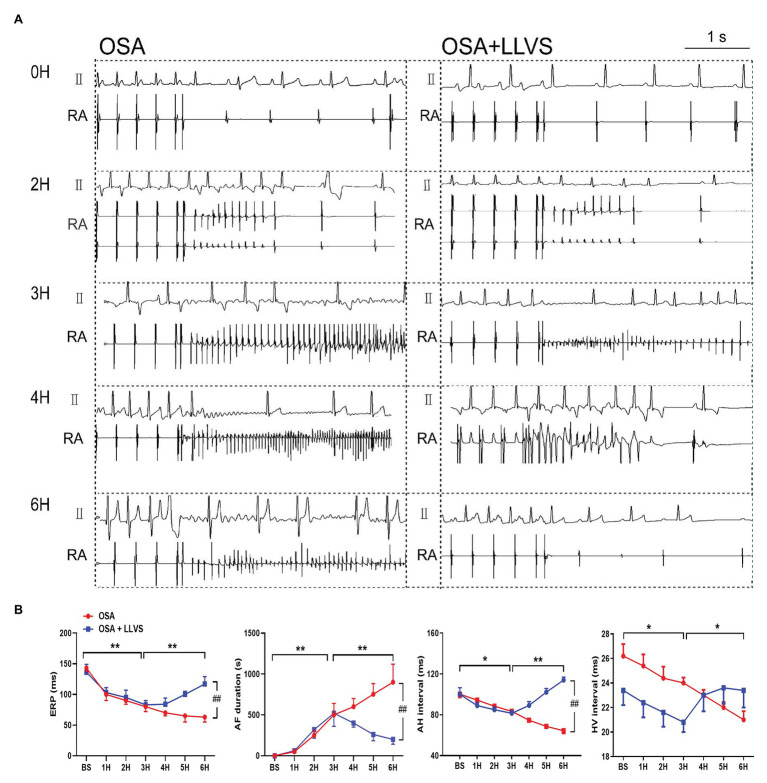
**(A)** Representative examples of AF episodes in the OSA and OSA+LLVS groups at baseline, the 2nd hour, the 3rd hour, the 4th hour, and the 6th hour. **(B)** Changes in ERP, AF duration, AH interval, and HV interval at each hour among the three groups. ^*^p<0.05, ^**^p<0.01, ^##^*p*<0.05; ERP, effective refractory period; AF, atrial fibrillation; AH, Atrial-His; HV, His-Ventricle; H, hours.

Compared with the baseline of the experiment, the AF duration, and SNRT were significantly increased. The atrial ERP, AH interval, and HV interval also gradually decreased over the 6h in the OSA group (all *p*<0.01), an analogous pattern also emerged in the first 3h in the OSA+LLVS group (all *p*<0.01), but after the 4th hour, the trends of all the parameters gradually reversed and returned to their baseline values (all *p*<0.01; Figures 3B, 5B). The representatives of AF and SNRT are shown in Figure 3A and Figure 5A, respectively.

Cx40 and Cx43 were detected through immunohistochemistry staining (Figures 3D,E). Compared with the control group, the expression levels of Cx40 and Cx43 were decreased in the OSA group, and LLVS treatment could increase expression.

These results demonstrate that the electrophysiological changes in the acute OSA-induced AF were consistent with AF features in humans and that the LLVS could reverse these changes.

### Effect of LLVS on the mRNA Expression Level of Ion Channels in Acute OSA

To clarify the changes in ion channels that are associated with AF, the qRT-PCR was administered (Figure 3C). The Nav1.5 channel was highly expressed in the OSA group, whereas its expression was lower in the OSA+LLVS group (*p*<0.01). The Cav1.2 channel was less expressed in the OSA group and the OSA+LLVS group than in the control group (*p*<0.01). The Kir2.1 channel and the Kir3.1 channel were highly expressed both in the OSA group and the OSA+LLVS group compared with those in the control group (*p*<0.01), and no significant difference was observed between the OSA group and the OSA+LLVS group (*p*>0.05). There was no significant difference in the Kir3.4 channel expression between the control and experimental group (*p*>0.05). Compared with that of the control group, the expression of the HCN4 channel in the OSA group and the OSA+LLVS group decreased (*p*<0.01), but there was no significant difference between the two groups.

### Effect of LLVS on Atrium Structural Remodeling in Acute OSA

**Figure 6:**
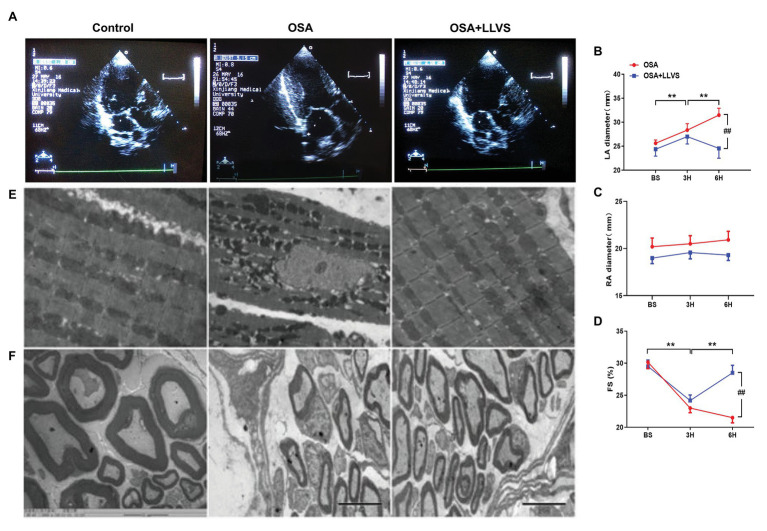
**(A)** Echocardiography shows images of the LA among the three groups. **(B)** The LA gradually and significantly increased after OSA, and the LA decreased through LLVS treatment. **(C)** There was no statistically significant difference in RA among the three groups. **(D)** OSA-induced FS gradually shorten in the OSA group, and LLVS treatment significantly increased FS. **(E)** Changes in the mitochondrial and stellate ganglion **(F)** by transmission electron microscopy among the three groups. ^**^p<0.05 vs. the control group; ^#^*p*<0.05 vs. the OSA+LLVS group; LA, left atrium; RA, right atrium; FS, fractional shortening; BS, baseline; H, hours.

Changes in the atrial structure were evaluated by echocardiography at baseline, in the 3rd hour and the 6th hour. In the OSA group, the LA gradually became swollen and spherical, deviating from its original oval shape, and the LA diameter significantly increased. This structural change was also represented in the OSA+LLVS group at the 3rd hour. After the LLVS treatment was administered from the 3rd hour to the 6th hour, the shape of the LA gradually changed from sphere to oval, and the LA diameter decreased (Figures 6A,B). Conversely, the RA showed no statistically significant difference among the three groups in the process of OSA (Figure 6C). Additionally, the fractional shortening (FS) of the left ventricular was also analyzed, and it was found that the FS was significantly shorter in the OSA+LLVS group than in the control group (*p*<0.05; Figure 6D).

Transmission electron microscopy was used to observe the ultrastructural changes of cardiomyocytes. We found that the mitochondria in cardiomyocytes evidenced swelling to different degrees in the OSA group compared to those in the control group, and LLVS intervention was able to attenuate the swelling of the mitochondria to a certain extent (Figure 6E).

**Figure 7:**
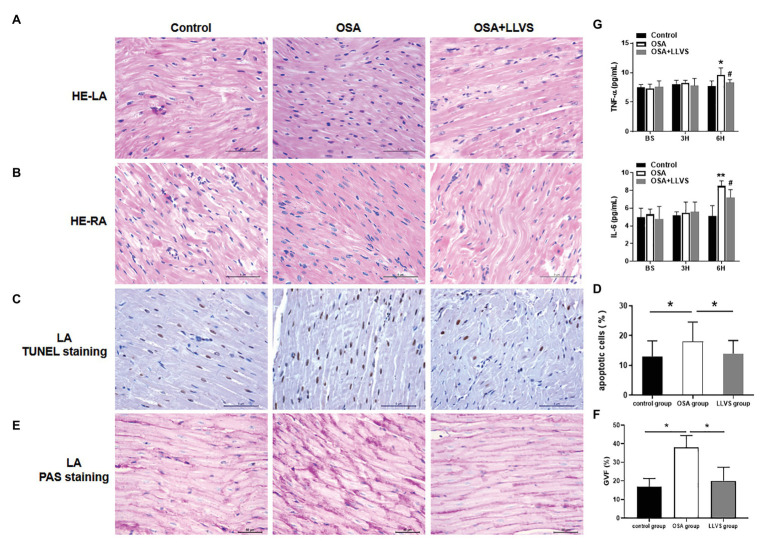
Changes in cardiomyocytes among the three groups. HE staining showed that cardiomyocytes in the LA **(A)** and RA **(B)** were arranged tightly and orderly across the three groups. **(C)** Representative TUNEL staining on the LA across the three groups. **(D)** Quantitative analysis of TUNEL-positive cells in the LA of each group. **(E)** Representative PAS staining in the LA among the three groups. **(F)** Quantitative analysis of the glycogen volume fraction in the LA of each group. **(G)** The concentrations of TNF-α and IL-6 among the three groups at baseline, the 3rd hour, and the 6th hour. ^*^p<0.05, ^**^p<0.01 vs. the control group; ^#^*p*<0.05 vs. the OSA+LLVS group; HE, hematoxylin and eosin; TUNEL, terminal deoxynucleotidyl transferase dUTP nick end labeling; PAS, periodic acid-Schiff; TNF-α, tumor necrosis factor alpha; IL-6, interleukin 6; H, hour.

To evaluate changes in myocardial tissues, HE staining was conducted. The atrial myocytes in LA and RA were arranged tightly and orderly in the three groups, and neither showed any significant difference (Figures 7A,B).

TUNEL staining was used to evaluate atrial cardiomyocyte apoptosis, the OSA group had more brown-stained nuclei compared with the control group, meaning that apoptosis in the OSA group was more serious, LLVS could attenuate cardiomyocyte apoptosis in the atria (Figures 7C,D).

PAS staining was conducted to evaluate glycogen deposition in atrial tissues. In contrast to that in the control group, glycogen deposition was not only observed at the junction of cardiomyocytes but also in cardiomyocytes in the OSA group. After LLVS treatment, glycogen deposition was attenuated (*p*<0.05; Figures 7E,F).

In conclusion, acute OSA-induced AF could change the structure of LA, and this remodeling could be reversed by LLVS treatment.

### Effect of LLVS on the Expression of Inflammatory Cytokines

ELISAs were used to detect the concentrations of TNF-α and IL-6. The concentrations of TNF-α and IL-6 in the OSA group were significantly increased compared to those in the control group, and these levels decreased in the OSA+LLVS group, indicating that LLVS could inhibit the expression of inflammatory cytokines (Figure 7G).

## Discussion

### Major Findings

This study had the following findings: (1) LLVS reversed OSA-induced vagosympathetic nerve overdriving, including the high expression of ChAT, TH, PGP9.5 and enhanced silver staining in LA tissues, as well as a high concentration of NE. Additionally, both parasympathetic and sympathetic activity increased significantly during apnea, and a high state of sympathetic activity after ventilation was observed. (2) LLVS increased ERP, decreased AF duration, and AF inducibility in the process of OSA-induced AF. The present study is the first to report prolonged SNRT, high expression of Cx43 and Cx40, and ion channel expression abnormality in the acute OSA-induced AF, which could be attenuated by LLVS treatment. (3) Systemic inflammation, glycogen deposition, cardiomyocyte apoptosis, and mitochondrial damage in LA cardiomyocytes were also reported in the acute OSA-induced AF model for the first time, whereas LLVS treatment weakened these harmful effects. In a word, LLVS intervention could attenuate the parameter changes mentioned above, and these effects may occur through modulating the ANS and systematic inflammation.

### LLVS Modulated ANS Activity in the Acute OSA Model

There is considerable evidence that ANS plays a crucial part in the initiation and maintenance of AF (Linz et al., 2012). In the present study, we found that the concentration of norepinephrine was significantly increased in the OSA group, as was the level of TH, indicating sympathetic activity was overdriving. Meanwhile, the levels of ChAT, which reflect the activity of the parasympathetic system were also increased. The present study also found that the expression level of PGP9.5 and silver staining was enhanced in the OSA group. This demonstrates that the distribution of nerve fibers was hyperinvernation and that nerve discharge also showed a high activity of ANS during apnea. These findings are consistent with other clinical and animal studies that have shown that both the sympathetic and parasympathetic systems were activated in the paroxysmal AF (Amar et al., 2003; Burashnikov and Antzelevitch, 2003; Patterson et al., 2005; Choi et al., 2010). We also found that the myelinated nerve fibers in the left stellate ganglion in the OSA group was swollen and degenerated, indicating nerve structural remodeling. After the LLVS intervention, both sympathetic and parasympathetic related parameters were attenuated. We also found that during apnea, HR and BP gradually decreased, as SNRT increased; once the apnea interval transitioned to ventilation, BP and HR rose sharply, indicating that the homeostasis of the sympathetic nerve and vagus nerve were undergoing dramatic changes that would promote the initiation of AF. Furthermore, the prolonged SNRT was significantly shortened after LLVS was administered, accompanied by lower AF inducibility. Taken together these results demonstrated that ANS overdriving played an important role in the occurrence of acute OSA-induced AF, while LLVS could suppress the inducibility of AF partly by regulating the imbalance of ANS.

### Effect of LLVS on the Level of Ion Channels mRNA Changes in Acute OSA-Induced AF

The present study revealed a change in ion channels in the acute-induced AF. As is widely known, ion channel abnormality plays a very important role in the processes underlying the occurrence and maintenance of AF. Several studies have reported that higher expression of Nav1.5 (Chatelier et al., 2012; Makara et al., 2014), Kir2.1 (Xia et al., 2005; He et al., 2006), Kir3.1, and Kir3.4 (Zhang et al., 2009; Bingen et al., 2013), and lower expression of HCN4 (Macri et al., 2014; Ishikawa et al., 2017) and Cav1.2 (Nakatani et al., 2013; Morishima et al., 2016) can be attributed to the initiation and maintenance of AF. In our present study, we evaluated the level of ion channels through detecting the gene expression with qRT-PCR, and found that a higher expression of Nav1.5 was manifested in the OSA group, followed by a decreased level in the OSA+LLVS group, which was in line with previous studies (Chatelier et al., 2012; Makara et al., 2014). At the same time, the higher gene expression levels of Kir2.1, Kir3.1, and a lower gene expression level of Cav1.2 were also represented in the OSA group, although these levels were not attenuated after LLVS was administered. No obvious change in Kir3.4 was observed between the control and the experimental groups. Furthermore, the gene expression level of HCN4 was lower in the OSA group, indicating that the current of the sinus atrial node was decreased, which could explain the SNRT prolongation observed. This also was consistent with previous reports that the lower current of HCN4 promotes the occurrence of AF (Macri et al., 2014; Ishikawa et al., 2017). However, the present study did not detect an increase in the level of HC4 after LLVS intervention. Combined with our experiment, we speculate that the reason for the inconsistency in the expression levels of ion channels with prior studies might be due to a shorter experiment time. Future studies with a much longer observation time are needed.

### LLVS Inhibited Electrical Remodeling in the Acute OSA Model

This study investigated changes in the electrical parameters of AF as a consequence of OSA. Previous research has shown that a shorter ERP is strongly associated with a high incidence of AF (Josephson, 1993). SNRT, reflecting the function of the sinus node, became longer in an aging mouse model, in parallel with increasing AF inducibility (Luo et al., 2013). Clinical studies have also indicated that a longer SNRT is an indicator of AF recurrence after catheter ablation (Yamaguchi et al., 2018). Both animal and human studies have demonstrated that a lengthy AF duration could affect and diminish the function of the AV node, which would manifest in longer AH and HV intervals (Zhang and Mazgalev, 2012; Sairaku et al., 2016). In our present study, we found that ERP was progressively and significantly decreased and that the AF duration time progressively increased during the 6h of OSA, in line with previous studies. For the first time, the present study reports gradually prolonged SNRT in the OSA. All of these changes indicate that the present OSA model could have induced changes in electrical parameters, such as those evident in AF. These findings indicate that this acute OSA-induced AF model was successfully established and could further provide a reliable model for the basic study of OSA. At the same time, we also found that these deteriorated parameters could be attenuated or reversed to baseline by administering LLVS treatment in the final 3h, indicating the crucial role of the ANS in the process of acute electrical remodeling. Moreover, heterogeneous myocardial conduction significantly increased, as shown by the increased expression of Cx40 and Cx43.

### LLVS Reversed Systemic Inflammation, Glycogen Deposition, and Cardiomyocyte Apoptosis Induced by OSA

Recently, increasing evidence has pointed to a role played by inflammatory agents in the initiation of AF (Hu et al., 2015). Serval clinical and animal studies have reported that atrial tissues in AF can be infiltrated with neutrophils and lymphocytes, in contrast to those in the sinus rhythm such as TNF-α and IL-6 (Frustaci et al., 1997; Patel et al., 2010; Guo et al., 2012; Smit et al., 2012; Jacob et al., 2014). The serum level of CRP could also be used to predict the risk of developing AF, suggesting that the presence of inflammatory cytokines could promote the initiation of AF (Aviles et al., 2003). In the present study, we showed that the concentrations of TNF-α and IL-6 significantly increased in conjunction with mitochondrial swelling and cardiomyocyte apoptosis, which is consistent with previous reports that the overexpression of inflammatory cytokines promotes cardiac myolysis and apoptosis, and that this effect could be reversed by specific inflammatory cytokine receptor blockers (Liao et al., 2010; Hoogstra-Berends et al., 2012). Recent studies of LLVS are well-known because of the multiple functions, in particular, its anti-inflammatory effects (Andersson and Tracey, 2012; Pavlov and Tracey, 2012; Stavrakis et al., 2015; Koopman et al., 2016). After intervention with LLVS the TNF-α and IL-6 concentrations were decreased, and simultaneously, there was a decreased incidence of AF. The present study also found glycogen deposition in cardiomyocytes in the OSA group, in response to the hypoxic environment, and a strong relationship between sugar metabolism and the inflammatory response was documented (Ma et al., 2020). Therefore, we hypothesize that the hypoxic environment promoted the high glycogen deposition, increased the high expression of inflammatory cytokines, and led to atrial structural remodeling. The mechanism of LLVS treatment inhibited the production of inflammatory cytokines and subsequently reversed the atrial structural remodeling. To the best of our knowledge, the concept of atrial structural remodeling, attributed to the maintenance of chronic AF is widely accepted (Zhao et al., 2014; Zhang et al., 2015b; Sun et al., 2017; Dai et al., 2019; Yang et al., 2019). However, the effect of acute OSA on cardiomyocytes is not fully understood. We found that the LA stretches, similar to the features of atrial changes caused by human OSA (Orban et al., 2008; Linz et al., 2011a,b; Valenza et al., 2014), which could be reversed by the LLVS. In our present study, mitochondrial swelling, glycogen deposition, and myocardial apoptosis were observed in the OSA group, in the absence of fibrosis and hypertrophy. This study is the first to report a change in atrial cardiomyocytes in the acute OSA-induced AF model. These findings indicate that structural remodeling also occurs in the acute process of OSA, and promotes the initiation and maintenance of AF.

### Possible Mechanisms of LLVS Reverse OSA-Induced AF

**Figure 8:**
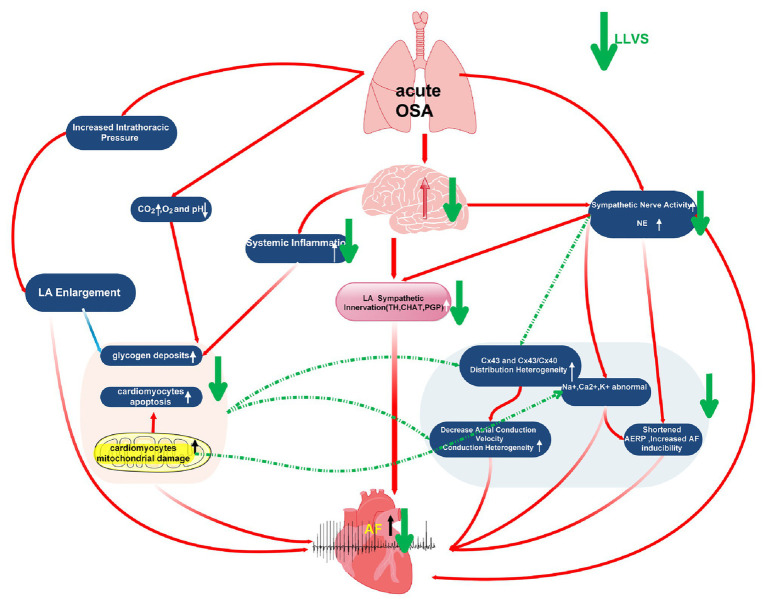
Graphic illustration of possible mechanism about acute OSA induced-atrial fibrillation.

In conclusion, by taking into account the results of the present study alongside our previous studies, we hypothesize the possible mechanisms as follows: (1) In apnea, thoracic expansion generates a negative intrathoracic pressure, which is subsequently transmitted to the thin-walled atrium and leads to LA stretching, which has been recognized as one of the highest risk factors of AF (Orban et al., 2008; Linz et al., 2011a,b; Valenza et al., 2014). (2) In the process of OSA, repeated apneas can lead to hypoxemia and hypercapnia, as well as secondary sympathetic nerve activation and systemic inflammation, both of which promote glycogen deposition, apoptosis, and mitochondrial damage in LA cardiomyocytes, which occur in parallel with abnormally expressed ion channels (including K^+^, Na^+^, Ca^2+^, and HCN4) and Cx43 and Cx40. All these changing parameters ultimately shorten the ERP, increase AF susceptibility and atrial conduction heterogeneity, promoting AF initiation and maintenance. In our present study, although hypocapnia and hypoxemia were not attenuated after LLVS was administered, the sympathetic neural hyperactivation was modulated, and the systemic inflammation was inhibited, also improving the remodeling of myocardial tissue structure, ion imbalance, and abnormal Cx43 and Cx40 expression, while ultimately inhibiting AF occurrence (Figure 8).

### Study Limitations

The present study has several limitations. First, the invasiveness of LLVS limits its clinical application. A noninvasive low-level transcutaneous auricular branch of the vagus nerve electrical stimulation has been shown to decrease AF inducibility (Sha et al., 2011; Yu et al., 2013, 2017; Stavrakis et al., 2015). Second, anesthesia inhibits autonomic nervous activity to some degree. However, all animals in the three groups underwent similar anesthesia, and the HR and BP parameters were stable in the control group, which may have neutralized the effect of the anesthesia. Third, we used qRT-PCR to determine the exact level of ion channels because of equipment and technology limitations. However, the most direct description of the ion current can be reflected by the patch-clamp technique. Thus, the next step will be to measure the exact level of ionic current by introducing patch-clamp techniques. Fourth, the present study only described the structural changes occurring only in the LA in detail, and the differences in other chambers were not investigated. Fifth, we only proposed the possible hypothetical mechanism of atrial fibrillation caused by OSA without further verification. Finally, only 6h of OSA and AF was induced in our study’s experiment, the relationship of chronic OSA and AF has not been investigated, and whether LLVS has long term anti-AF properties or whether either can reverse the effects of atrial remodeling in chronic OSA warrants further exploration in future studies.

## Conclusion

In summary, this study found that OSA induced sympathetic overactivity, enhanced LA sympathetic innervation and systematic inflammation, myocardial mitochondrial swelling, glycogen accumulation, and myocardial apoptosis. We hypothesize that both neural and structural remodeling promoted ion abnormalities, Cx43/Cx40 disturbances, and conduction heterogeneity. Since all of these effects could be attributed to the initiation and maintenance of AF, the present study indicates that LLVS could reverse the related parameters to a degree. This approach might therefore provide a novel option and potential therapeutic target for AF in acute OSA.

## Data Availability Statement

The raw data supporting the conclusions of this article will be made available by the authors, without undue reservation.

## Ethics Statement

The animal study was reviewed and approved by the Animal Use and Management Ethics Committee of the First Affiliated Hospital of Xinjiang Medical University.

## Author Contributions

YG, JX, QM, and HS contributed to the animal experiments. YG and LZ contributed to the statistical analysis and interpretation. GC, XZ, LZ, and BT contributed to the funding acquisition, conception, and design of the study. All authors contributed to the writing, critical reading, and approval of the manuscript.

### Conflict of Interest

The authors declare that the research was conducted in the absence of any commercial or financial relationships that could be construed as a potential conflict of interest.
